# Further evidence of an excess of risk of pleural malignant mesothelioma in textile workers in Prato (Italy).

**DOI:** 10.1038/bjc.1991.311

**Published:** 1991-08

**Authors:** E. Paci, M. Zappa, L. Paoletti, E. Buiatti, E. Chellini, E. Merler, A. Seniori Costantini

**Affiliations:** Centro per lo Studio e la Prevenzione Oncologica, Unit of Epidemiology, Florence, Italy.


 © Macmillan Press Ltd., 1991

### Further evidence of an excess of risk of pleural malignant mesothelioma in textile workers in Prato (Italy)

E. Paci[1], M. Zappa[1], L. Paoletti[2], E. Buiatti[1], E. Chellini[1], E. Merler[1] & A. Seniori Costantini[1]

[1]*Centro per lo Studio e la Prevenzione Oncologica, Unit of Epidemiology, Florence;* [2]*Istituto Superiore di Sanità, Ultrastructure Laboratory, Rome, Italy.*

An epidemiological study (Paci *et al.*, 1987) showed that among thirteen pleural malignant mesothelioma (PMM) cases incident in the Province of Florence during the years 1979-1984, there were six cases who had been workers in the textile industry in the Prato area, five of them in a particular job, the sorting of rags. In this area of the Province of Florence (population 206,205 in 1981), there is a high concentration of textile factories (11,192 in 1981) with a total of 48,225 employees. This is more than 20% of all those employed in manufacturing industry in the entire province (population 1,199,988) and about 70% of those employed in the textile industry (ISTAT, 1981). This finding was unexpected, as until that time there had been no known use of asbestos fibres in that particular production cycle.

A subsequent environmental hygiene survey (Quinn *et al.*, 1987) showed that the Prato textile workers were exposed to asbestos through the recycling of bags which had contained asbestos fibres. This 'improper' use had begun during the years following the Second World War and had continued, although in a reduced form, until the period of the environmental study (1985).

Already diagnosed PMM and all cases of pleural cancerous infiltration between 1970 (the first year available) and 1988 were taken from the archives of the Department of Pathology of the University of Florence (Director S. Dini) and reexamined. The vital status of each subject was determined and, if deceased, the nearest living relative was identified. All cases of their relatives were interviewed by trained nurses using a structured questionnaire on smoking habits and work history; in addition, the case or the substitute was expressly asked if he/she had any knowledge of having worked with material containing asbestos and if there were any known factories using asbestos near his/her place of residence. Non-occupational sources of exposure to asbestos were also investigated.

A total of 61 cases of PMM (44 males, 17 females) occurred in the period 1970-1988 among residents in the Province of Florence. In one case the interview was refused, in another it was not possible to trace the living relatives. Out of the 61 PMM cases, 22 (16 males, six females) ever-worked as textile workers and out of these 16 (14 males, two females) as rag sorters. The first textile PMM case occurred in 1973, but since then until 1988 textile cases have regularly appeared. The mean age at first exposure was 31.6 years (s.d. ± 11.0; range 18-55) and the mean duration of work in the textile industry was 24.4 years (s.d. ± 9.8; range 9-38). The average latency (time between first exposure and year at diagnosis) for these subjects was 29.8 (s.d. ± 8.1; range 10-40).

The determination of asbestos fibres present in samples of pleural or lung tissue was carried out at the laboratory of the Italian National Health Institute (Istituto Superiore di Sanità, Rome) by means of the high resolution electron microscope (TEM). Sufficient material was available for fibre analyses in only two samples of lung tissue: the asbestos fibre concentration in the lung parenchyma was 2,387 ff $mg^{-1}$ (crocidolite) fibres in one case and 10,146 ff $mg^{-1}$ (crocidolite and chrysotile) in the other. A value of 1,000 ff $mg^{-1}$ in the lung tissue was assumed as indicative of occupational exposure to asbestos (Mowe *et al.*, 1984; Paoletti *et al.*, 1987).

Table I shows the distribution of the longest-held and ever worked job-titles among textile cases and confirms the relevance of the rag sorter job title among cases. An industrial hygiene survey led to the identification of three sources of asbestos in this textile industry (Quinn *et al.*, 1987):

(a) the use of jute and polypropylene bags which had once contained asbestos to wrap rags once they had gone through the first rag sorting phase;
(b) the release of asbestos fibres during the tearing-up phase of army uniforms and other war materials;
(c) the addition of asbestos-chrysotile fibres to woollen yarn during the spinning phase.

The first form of exposure was still going on (although in a reduced form) at the time of the environmental survey in 1985; the other two refer to periods in the past. The tearing up of army uniforms containing asbestos occurred in the years immediately following the Second World War, while the addition of asbestos-chrysotile in spinning lasted for only a few years in the 1970's and involved primarily the later phases of the production cycle, rather than the sorting of rags which in Prato is the first step in reweaving woollen cloth from old woollen garments. Six out of the 22 textile cases worked only in the last phases of the production process (spinning, weaving and dying), supporting a possible exposure to asbestos in other phases of the textile production process.

The results of this research confirm the previously reported high incidence of malignant mesotheliomas among textile workers in Prato (Florence) and in particular among rag sorters. This risk was unexpected and the finding should alert us to the possible occurrence of asbestos-related tumour epidemics from unknown and unexpected sources of pollution resulting from industrial production processes (Talcott *et al.*, 1989).

**Table I** Longest-held and ever worked job titles of the textile workers PMM cases*

| Job titles | Males LH | Males EW | Females LH | Females EW | Total LH | Total EW |
|---|---|---|---|---|---|---|
| Rag-sorting | 10 | 14 | 2 | 2 | 12 | 16 |
| Spinning | – | 3 | – | – | – | 3 |
| Weaving | 1 | 1 | 2 | 3 | 3 | 4 |
| Dyeing | 1 | 2 | – | – | 1 | 2 |
| Other | 1 | 2 | 2 | 2 | 3 | 4 |

LH, Longest-held job title; EW, ever-worked job title; *each subject may have worked at more than one job.

We thank Patrick Johnson for his linguistic support in the preparation of this paper.

Correspondence: E. Paci, Centro per lo Studio e la Prevenzione Oncologica, Unit of Epidemiology - Viale Volta 171, 50125 Florence, Italy.
Received 19 July 1990; and in revised form 21 March 1991.

### References

ISTAT (1981). Italian National Insitute of Statistics – Censimento industria, commercio e artigianato 1981.

MOWE, G., GLYSETH, B., HARTVEIT, F. & SKANG, V. (1984). Occupational asbestos exposures, lung fiber concentration and latency time in malignant mesothelioma. *Scand. J. Work Environ. Health*, **10**, 293.

PACI, E., DINI, S., BUIATTI, E. & 3 others (1987). Malignant Mesothelioma in non asbestos textile workers in Florence. *Am. J. Indust. Med.*, **11**, 255.

PAOLETTI, L., BATISTI, D., CAIAZZA, S. & 6 others (1987). Mineral particles in the lungs of subjects resident in the Roma area and not occupationally exposed to mineral dust. *Environ. Res.*, **44**, 18.

QUINN, M., KRIEBEL, D., BUIATTI, E. & 4 others (1987). An asbestos hazard in the reprocessed textile industry. *Am. J. Indust. Med.*, **11**, 255.

TALCOTT, J.A., THURBER, W.A., ARLENE, R.N. & 5 others (1989). Asbestos associated diseases in a cohort of cigarette filter workers. *N. Engl. J. Med.*, **321**, 1220.


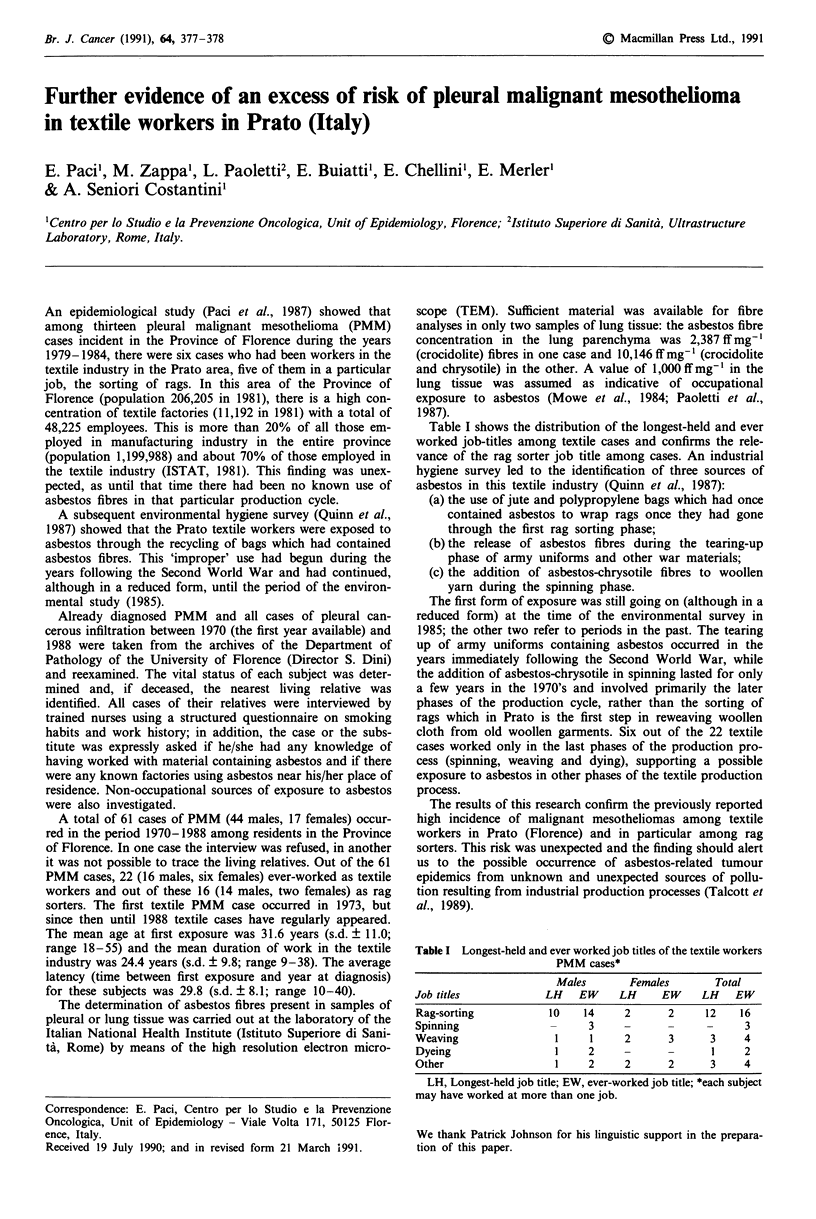

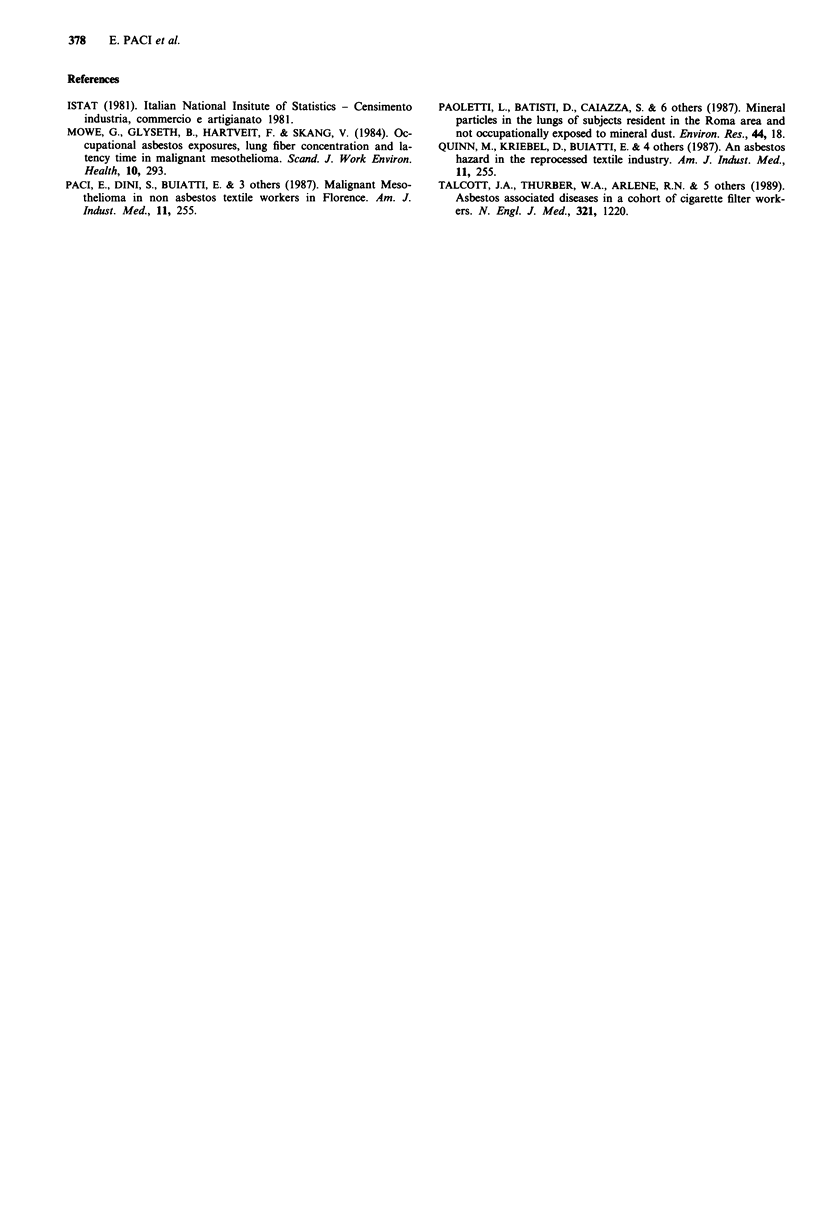


## References

[OCR_00159] Mowé G., Gylseth B., Hartveit F., Skaug V. (1984). Occupational asbestos exposure, lung-fiber concentration and latency time in malignant mesothelioma.. Scand J Work Environ Health.

[OCR_00165] Quinn M. M., Kriebel D., Buiatti E., Paci E., Sini S., Vannucchi G., Zappa M. (1987). An asbestos hazard in the reprocessed textile industry.. Am J Ind Med.

[OCR_00179] Talcott J. A., Thurber W. A., Kantor A. F., Gaensler E. A., Danahy J. F., Antman K. H., Li F. P. (1989). Asbestos-associated diseases in a cohort of cigarette-filter workers.. N Engl J Med.

